# Impact of bariatric surgery in elderly patients with obesity

**DOI:** 10.1590/0100-6991e-20223299-en

**Published:** 2022-07-08

**Authors:** ADRIANO F. PEREIRA, FERNANDO SANTA-CRUZ, LUCAS R. COUTINHO, MARIA CLARA P. T. VIEIRA-DE-MELO, EDUARDA A. HINRICHSEN, LUCIANA T. SIQUEIRA, JOSÉ-LUIZ FIGUEIREDO, ÁLVARO A. B. FERRAZ

**Affiliations:** 1 - Universidade Federal de Pernambuco, Pós-Graduação em Cirurgia - Recife - PE - Brasil; 2 - Universidade Federal de Pernambuco, Curso de Medicina - Recife - PE - Brasil; 3 - Hospital Getúlio Vargas, Programa de Residência Médica em Cirurgia Geral - Recife - PE - Brasil; 4 - Universidade Federal de Pernambuco, Departamento de Cirurgia - Recife - PE - Brasil

**Keywords:** Obesity, Bariatric Surgery, Elderly, Postoperative Complications, Obesidade, Cirurgia Bariátrica, Idosos, Complicações Pós-Operatórias

## Abstract

**Introduction:**

to evaluate the long-term impact of bariatric surgery in the elderly population.

**Methods:**

a retrospective study including all patients older than 60 years who underwent Roux-en-Y gastric bypass (RYGB) at our center and maintained a follow-up longer than 1 year. Clinical and laboratory variables were studied to assess remission of obesity and its comorbidities, as well as variables directly related to the surgical procedure itself, including early and late complications.

**Results:**

fifty-six patients were studied, mostly female (76,8%), with a mean age of 64.02 ± 3.34. A rate of complications of 37,5% was observed, with 10,7% requiring hospital admission and emergency surgery. The mean excess weight loss (%EWL) was 74.22% ± 26.76. The remission rates of hypertension and diabetes mellitus were 26.08% and 54.54%, respectively. There was significant difference in BMI reduction (12.25 ± 5.42, p<0.001), total cholesterol (31.37 ± 38.89 p<0,001), LDL cholesterol (23.45 ± 34.9, p=0.002), HDL cholesterol (5.14 ± 11.13, p=0,024), triglycerides (48.85 ± 56.15 p<0.001), HbA1C (1,81 ± 1,97, p<0,001) e PCR (1.43 ± 1.96, p<0.001).

**Conclusion:**

bariatric surgery was effective in weight loss and remission of comorbidities in the elderly obese population within the long term.

## RESUMO

**Introdução:**

avaliar o impacto da cirurgia bariátrica na população idosa no longo prazo.

**Métodos:**

estudo retrospectivo que incluiu todos os pacientes com mais de 60 anos submetidos ao Bypass gástrico em Y de Roux (BGYR) em nosso centro e que mantiveram seguimento superior a 1 ano. Foram estudadas variáveis clínicas e laboratoriais para avaliação da remissão da obesidade e suas comorbidades, além de variáveis diretamente relacionados ao procedimento cirúrgico em si, incluindo complicações precoces e tardias.

**Resultados:**

foram estudados 56 pacientes, a maioria do sexo feminino (76,8%), com idade média de 64,02 ± 3,34. Foi observada uma taxa de complicações de 37,5%, com 10,7% requerendo internamento hospitalar e cirurgia de urgência. A média geral da perda do excesso de peso (%PEP) foi de 74,22% ± 26,76. As taxas de remissão de hipertensão e diabetes mellitus foram de 26,08% e 54,54%, respectivamente. Houve variação significativa no IMC (12,25 ± 5,42, p<0,001), colesterol total (31,37 ± 38,89 p<0,001), colesterol LDL (23,45 ± 34,9, p=0,002), colesterol HDL (5,14 ± 11,13, p=0,024), triglicerídeos (48,85 ± 56,15 p<0,001), HbA1C (1,81 ± 1,97, p<0,001) e PCR (1,43 ± 1,96, p<0,001).

**Conclusão:**

a cirurgia bariátrica se mostrou eficaz na perda de peso e na remissão de comorbidades na população de idosos com obesidade no longo prazo..

## INTRODUCTION

Along with the general population, elderly individuals have also shown a vertiginous acceleration in obesity rates. In the United States, the prevalence of Body Mass Index (BMI) greater than or equal to 30kg/m² in people over 60 years of age increased from 23.6% in 1990 to 37.4% in 2010^1^. Aside from the multi-systemic repercussions of obesity as in the general population, older adults with this condition are also more prone to disability. In this sense, in addition to developing musculoskeletal diseases more frequently and earlier, excess weight in these patients significantly contributes to the worsening of problems directly related to morbidity and mortality, such as postural instability and risk of falls^2^ ^,^ ^3^.

Concomitantly with the global scenario of obesity, the elderly population has shown significant growth in absolute and relative terms worldwide. This phenomenon, related to the reduction of mortality and birth rates, was initially experienced by developed countries and, later, by developing ones, as is the case of Brazil. The aging process is linked to challenges from a social security point of view and especially from a public health perspective, given the predisposition of this age group to different clinical conditions, such as chronic and malignant diseases^4^ ^,^ ^5^. Thus, the already described increase in the prevalence of obesity among the elderly brings up discussions about the management of this condition in such patients, since there is a finer line between risks and benefits in this scenario^2^.

Within this perspective, bariatric surgery and its various techniques have been recently studied in the referred population to verify if there is a solidity of results like those of the procedures performed in younger patients. Several studies have already demonstrated the safety of such operations in the elderly, with perioperative morbidity and mortality rates like the ones of the general population, both in relation to RYGB and SG6-8. On the other hand, when it comes to individuals over 65 years of age, there is also evidence of higher rates of complications and less weight loss, but the surgery is still considered safe^9^ ^,^ ^10^. Regarding the long-term clinical relevance, despite a smaller number of prospective studies, there are indications of benefits in comorbidities control^11^ ^,^ ^12^.

Given this situation, we should note that surgical procedures to treat obesity in the elderly have shown a growing trend, even without formal endorsement by the responsible societies and bodies. Thus, our objective with this study was to evaluate the impact of bariatric surgery on the elderly population, highlighting its risks and benefits.

## METHODS

### Study design and population

We conducted a longitudinal, retrospective, cohort study at the Hospital das Clínicas of UFPE during the period from June 2021 to January 2022. The study included patients of both sexes aged 60 years or older with formal indication for bariatric surgery, operated by the RYGB technique in our center between 2003 and 2018. We excluded from the analysis patients with a history of neoplasia in the previous five years, with high preoperative risk, undergoing SG, or with a postoperative follow-up of less than one year.

### Ethical procedures

The work was approved by the Ethics Committee for Research on Human Beings of the Health Sciences Center at UFPE (CAAE: 50728.121.1.0000.5208). The researcher followed all the principles that govern the current Medical Ethics Code, in Resolution 466/12 of the National Health Council - CONEP.

### Data collection and statistical analysis

Data were collected from the researcher’s own electronic platform. According to the type of surgery, the observed variables were sex, medication use, diabetes mellitus (DM), systemic arterial hypertension (SAH), and follow-up time. We compared the variables between preoperative and late postoperative periods, as follows: HbA1c, CRP, fasting glucose, serum insulin, serum C-peptide, HDL and LDL cholesterol, triglycerides, iron, vitamin B12, zinc, vitamin D, albumin, presence of comorbidities, medication use, and BMI. The study of complications included occurrence of severe iron deficiency anemia, hospitalization, need for urgent surgery, anastomotic ulcer, and death.

We described categorical variables as absolute frequencies and percentages. For numerical ones, we used mean, standard deviation (mean ± SD), median, and 25^th^ and 75^th^ percentiles (median (P25; P75)). We used the paired Student’s t-test to compare the difference between the assessments (variation) when the variation was normally distributed, and the paired Wilcoxon test when normality was rejected. We verified normality with the Shapiro-Wilk test and assessed the equality of variances with the Levene’s F test. The margin of error used in the decision of the statistical tests was 5%. The data were entered into an EXCEL^®^ spreadsheet and the software used for the statistical calculations was the IBM^®^ SPSS, version 25.

## RESULTS

The age of the 56 patients analyzed ranged from 60 to 73 years, with a mean of 64.02 years, standard deviation of 3.34 years, and median of 63.5 years. Table 1 shows the general characteristics of the included patients. Female sex predominated, 76.8%; the presence of DM was 58.9%; 83.93% of the patients used continuous medication; 82.1% had SAH and 48.2% had a postoperative follow-up between one and five years.

**Table 1:** General characteristics.

Variable	RYGB n (%)
Sex	
Male	13 (23.2)
Female	43 (76.8)
DM	33 (58.9)
HAS	46 (82.1)
Follow-up time	
1-5 years	27 (48.2)
>5 years	29 (51.8)

^1^Fisher’s exact test.

Table 2 presents the postoperative complications. Among the 56 studied patients, 21 had complications in the postoperative period: six cases of anastomotic ulcer, six cases of Petersen’s hernia, and nine cases of severe iron deficiency anemia, requiring venous iron replacement. Of the six cases that required urgent surgery, two were due to acute cholecystitis, three due to intestinal obstruction secondary to Petersen’s hernia, and one due to refractory upper gastrointestinal bleeding.

**Table 2:** Surgical complications.

Variable	RYGB n (%)
Complications	21 (37.5)
Urgent surgery	6 (10.7)
Admission	6 (10.7)

^1^Fisher’s exact test.

As shown in Tables 3 and 4, there was a reduction in BMI and in the laboratory parameters CRP, HbA1C, fasting glucose, serum insulin, C-Peptide, triglycerides, iron, and albumin. The difference was statistically significant regarding the variation of BMI (12.25 ± 5.42, p<0.001), CRP (1.43 ± 1.96, p<0.001), HbA1C (1.81 ± 1.97, p<0.001), fasting blood glucose (31.22 ± 45.84, p<0.001), serum insulin (14.99 ± 14.78, p<0.001), C-Peptide (0.74 ± 1.66, p=0.028), and triglycerides (48.85 ± 56.15 p<0.001).

**Table 3:** BMI and laboratory parameters.

		Assessment			
		Preoperative	Postoperative	p-value	Absolute difference
Variable	n	Mean ± SD	Mean ± SD		Mean ± SD
		Median (P25; P75)	Median (P25; P75)		Median (P25; P75)
BMI	56	42.12 ± 5.78	29.87 ± 5.33	p1<0.001*	12.25 ± 5.42
		40.91 (37.40; 44.99)	29.31 (26.61; 31.60)		11.72 (8.73; 15.23)
CRP	56	2.27 ± 2.21	0.83 ± 0.92	p2<0.001*	1.43 ± 1.96
		1.56 (0.30; 3.41)	0.52 (0.10; 1.30)		0.58 (0.05; 2.66)
HbA1c	56	7.63 ± 2.13	5.79 ± 0.80	p2<0.001*	1.81 ± 1.97
		6.60 (5.80; 9.60)	5.50 (5.30; 6.00)		1.15 (0.38; 3.25)
Glucose	56	128.74 ± 49.99	97.52 ± 25.30	p2<0.001*	31.22 ± 45.84
		109.00 (95.00; 145.00)	89.00 (84.00; 104.00)		13.00 (7.00; 37.00)
Insulin	56	22.52 ± 14.96	7.53 ± 4.42	p2<0.001*	14.99 ± 14.78
		16.00 (12.00; 34.00)	6.50 (5.00; 9.00)		10.60 (5.00; 22.50)
C-Peptide	56	3.09 ± 1.80	2.34 ± 1.03	p1=0.028*	0.74 ± 1.66
		2.64 (2.10; 4.13)	2.10 (1.50; 2.65)		0.60 (-0.25; 1.71)
Total Cholesterol	56	189.15 ± 24.98	157.78 ± 34.49	p1<0.001*	31.37 ± 38.89
		194.00 (170.00; 211.00)	163.00 (127.00; 176.00)		28.00 (-6.00; 65.00)
HDL	56	47.94 ± 11.43	53.08 ± 16.36	p1=0.024*	-5.14 ± 11.13
		Assessment			
		Preoperative	Postoperative	p-value	Absolute difference
		45.00 (40.00; 58.00)	50.00 (42.00; 67.00)		-6.00 (-13.00; 3.00)
LDL	56	108.87 ± 27.86	85.41 ± 27.06	p1=0.002*	23.45 ± 34.90
		112.80 (87.00; 128.20)	82.00 (66.00; 99.00)		23.00 (-10.80; 58.10)
Triglycerides	56	155.63 ± 68.31	106.78 ± 45.70	p2<0.001*	48.85 ± 56.15
		150.00 (111.00; 188.00)	108.00 (74.00; 125.00)		49.00 (13.00; 73.00)

*Significant difference at the 5.0% level; ^1^Paired Student t-test; ^2^Paired Wilcoxon test; ^3^Student’s t-test with equal variances; ^4^Mann-Whitney test; ^5^Student’s t-test with unequal variances.

**Table 4:** Micronutrients.

		Assessment			
		Preoperative	Postoperative	p-value	Absolute difference
Variable	n	Mean ± SD	Mean ± SD		Mean ± SD
		Median (P25; P75)	Median (P25; P75)		Median (P25; P75)
Iron	56	97.33 ± 27.97	97.30 ± 27.06	p1=0.995	0.04 ± 29.66
		98.00 (74.00; 117.00)	100.00 (76.00; 119.00)		2.00 (-20.00; 22.00)
B12 vitamin	56	632.80 ± 771.23	604.36 ± 506.15	p2=0.455	28.45 ± 950.67
		477.00 (342.00; 581.00)	464.00 (345.20; 695.30)		33.00 (-74.00; 178.00)
Zinc	56	316.31 ± 455.84	125.32 ± 208.37	p2=0.026*	190.99 ± 468.52
		84.70 (79.30; 457.30)	89.10 (54.75; 95.60)		20.10 (-4.00; 87.56)
D vitamin	5	23.38 ± 17.39	26.52 ± 7.29	p2=0.813	-3.14 ± 15.95
		24.60 (6.25; 39.90)	25.60 (21.00; 32.50)		-13.90 (-14.75; 13.85)
Albumin	27	4.02 ± 0.39	3.94 ± 0.39	p1=0.426	0.08 ± 0.54
		4.00 (3.80; 4.30)	4.00 (3.80; 4.20)		0.01 (-0.30; 0.32)

Significant difference at the level of 5.0%; ^1^Paired Student t-test; ^0^Paired Wilcoxon test; ^3^Student’s t-test with equal variances; ^4^Mann-Whitney test; ^5^Student’s t-test with unequal variances.

The mean values of total cholesterol, LDL, vitamin B12, and zinc decreased when comparing the pre and postoperative periods. The variation was statistically significant for total cholesterol (31.37 ± 38.89, p<0.001), LDL cholesterol (23.45 ± 34.9, p=0.002), and zinc (190.99 ± 468.52, p=0.026). When evaluating HDL cholesterol and vitamin D, we found an increase when comparing the pre and postoperative means. There was a statistically significant difference only in HDL cholesterol (5.14 ± 11.13, p=0.024).

As for the effectiveness of the procedure, we found that the general average of excess weight loss (%EWL) was 74.22% ± 26.76, ranging from 5.68% to 153.76%. When comparing the prevalence of DM in the pre and postoperative periods, we observed that of the 33 (58.9%) patients with DM initially, 15 (26.8%) remained diabetic after the procedure and of these, three (20%) showed attenuation of the disease. Therefore, the remission rate was 54.54%. Regarding SAH, we found that of the 46 (82.1%) hypertensive individuals in the preoperative period, 34 (60.71%) maintained the need of antihypertensive drugs, resulting in an absolute remission rate of 26.08%.

## DISCUSSION

Among the 62 patients eligible for the study, we included 56 in the analysis according to inclusion criteria; the others did not meet the minimum follow-up period. The methodology was designed and followed to assess the impact of bariatric surgery in the population over 60 years of age with obesity, as well as the possible associated risks, since there is still no consensus on the indication of the procedure in that population^10^ ^,^ ^13^ ^-^ ^16^.

Regarding the safety of bariatric surgery in the elderly, it is worth noting that the occurrence of postoperative complications is described between 12-42% in the literature^17^ ^,^ ^18^. In the present study, the complication rate was 37.5%. This relatively high incidence of complications may have been due to the greater coverage of conditions considered as complications when compared with other studies. Another contributing factor to this result was the exclusive analysis of RYGB, which is associated with a higher incidence of negative outcomes in this population, as seen by Xu et al. in their meta-analysis and Pajecki et al. in a randomized clinical trial. Both studies demonstrated that the SG technique had a lower complications rates in elderly patients when compared with RYGB^12^ ^,^ ^19^.

Within this perspective, mortality, both perioperative and late, is a very relevant parameter in the analysis of the safety of a surgical procedure. Susmallian et al. and Morgan et al. did not register deaths in the first 30 days in their respective cohorts, the latter demonstrating the absence of a statistically significant difference in long-term, all-cause mortality^18^ ^,^ ^20^. Sugerman et al. found no perioperative deaths, but there were 11 deaths (13.8%) in the late follow-up^8^. In line with the data presented, we did not observe any deaths in the postoperative period^7^.

The number of hospital admissions after the initial discharge from the surgical procedure is also a relevant aspect to be considered within the postoperative complications. In a randomized clinical trial with 18 subjects undergoing RYGB, Pajecki et al. found only one (5.6%) readmission^7^. In contrast, Moon et al. demonstrated, through a retrospective analysis, a considerably higher rate of readmissions in the group undergoing RYGB (13.8%), which was also suggested by and Xu et al.^6^ ^,^ ^19^. In agreement with the data from those two studies, we found six (10.71%) readmissions. We should note, however, that the longer follow-up time of our study compared with that of Pajecki et al. may have contributed to the higher rate in the total sample.

As for postoperative weight loss, there is still no clear consensus regarding the benefit of bariatric surgery in the elderly population. Giordano et al. conducted several studies on the topic, with conflicting results. In their systematic review of 26 studies, they found a combined mean %EWL of 53.8% in individuals over 60 years of age, comparable to that of younger people. However, the same author in a subsequent systematic review and meta-analysis showed a higher %EWL in patients younger than 60 years (mean difference of -7.34), with a statistically significance (p=0.007)^14^ ^,^ ^21^ ^,^ ^22^. With the same aim of correlating age group and %EWL after the procedure, Marczuk et al. and Lynch et al. published meta-analyses with nine and eighteen studies, respectively, both verifying a decrease in the effectiveness, in terms of weight loss, of bariatric surgery at more advanced ages^16^ ^,^ ^23^. In contrast, Keren et al. recorded, through a retrospective analysis, a higher mean %EWL in the group aged over 55 years (53.6%) compared with the groups aged between 35 and 55 years (45.6%) and under 35 years (41.3%)^24^. Despite the divergence in the literature, our data showed a significant %EWL, with a mean of 74.22% (± 26.76). This result is comparable to that of some existing publications, such as those by Lynch et al. and Susmallian et al., in which the follow-up period used was one year^16, 20^. 

Comorbidities remission is a fundamental parameter in defining the effectiveness of bariatric surgery, since the presence of DM, SAH, and dyslipidemia influence the indication of the procedure. Haywood et al. performed a systematic review of 69 studies on the treatment of obesity in patients aged 60 years or older. They analyzed the rate of DM remission after bariatric surgery and found that the definition of disease remission varies with the studies and, therefore, the resolution rate also varies widely, from 22% to 100%^25^. Giordano et al. described a resolution rate of 54.5%, while Susmallian et al. showed that 40.7% of patients with DM in the preoperative period showed remission^20^ ^,^ ^21^. In the present study, we considered as cured patients who stopped using hypoglycemic medication, the DM remission rate thus being 54.54%. This result differs from that presented by Susmalian et al., despite the diagnostic criteria and the mean patients’ age were similar in both studies. This may have been due to the longer follow-up time in our research.

Several studies evaluated the resolution of SAH, a condition established as a marker of cardiovascular risk. Marczuk et al. performed a meta-analysis of nine studies, and Giordano et al., a series of studies, two meta-analyses with seven and 11 publications, which showed that the rate of remission of SAH is lower in elderly patients undergoing bariatric surgery when compared with younger individuals^14^ ^,^ ^22^ ^,^ ^23^. Despite this, the elderly population still has a significant rate of SAH resolution, ranging from 20.25% to 57.93%^20^ ^,^ ^21^ ^,^ ^25^. Xu et al. showed in their meta-analysis that patients undergoing RYGB had an overall SAH remission rate of 25.64%^19^ ^,^ ^24^. Our findings agree with those described above, with a SAH remission of 26.08%.

That said, it is essential to highlight that, even though the present work was prepared to verify and discuss the variables previously explored, it is a retrospective cohort. In this sense, as it is not a prospective analysis, it is subject to a series of limitations and biases inherent to the type of study. In addition, there was no control group in which individuals under 60 years of age could have the same parameters evaluated and compared with the elderly population.

## CONCLUSION

Based on our results, it is possible to state that bariatric surgery proved to be effective in weight loss and in the remission of comorbidities in the elderly population with obesity, both in the short and long terms. Also, in accordance with existing references, the safety of this type of procedure for this population was ratified.

Finally, to obtain more solid conclusions about the present results, randomized controlled clinical trials are needed, with larger samples and prospectively obtained data to determine the real impact, safety, and benefits of this surgery in the referred population.
